# Effectiveness of a School-Based Oral Health Promotion Program on Dental Caries Among Iraqi School Children: A Cluster Randomised Controlled Trial

**DOI:** 10.1016/j.identj.2024.07.1214

**Published:** 2024-09-24

**Authors:** Hadi Ghasemi, Hanan Fadhil Alautry, Mohammed Hossein Khoshnevisan, Mahshid Namdari

**Affiliations:** a Department of Community Oral Health, School of Dentistry, Shahid Beheshti University of Medical Sciences, Tehran, Iran; b Department of Pediatric and Preventive Dentistry, School of Dentistry, Wasit University, Wasit, Iraq; c Dental Research Centre, Research Institute of Dental Sciences, Shahid Beheshti University of Medical Sciences, Tehran, Iran; d Department of Biostatistics, School of Allied Medical Sciences, Shahid Beheshti University of Medical Sciences, Tehran, Iran

**Keywords:** Fluoride, Oral health education, Schoolchildren, Cluster randomised controlled trial, Promotion program, Dental caries

## Abstract

**Aim:**

To assess the effectiveness of a school-based oral health promotion program on dental caries of permanent dentition among Iraqi children.

**Methods:**

A cluster randomised controlled trial was conducted with a parallel study group, comprising 8-10-year-old schoolchildren, 186 in each group. At the beginning of the study, subjects in the intervention group received oral health education and a single dose of 5% sodium fluoride varnish for all teeth surfaces while the control group only received oral health education. The primary outcome data in this study were caries increment and incidence after six months. The secondary outcome data was any change in oral health behaviors in the students of both groups after 3 months. The caries status was recorded according to International Caries Detection and Assessment System (ICDAS). Statistical analyses included the Chi-square test, McNemar test, independent t-test, simple and multiple logistic regression models.

**Results:**

Study participants included 372 children with no significant difference in baseline characteristics between intervention and control groups. An increase was evident in the mean scores of DMFS, DMFT, number of children with DMFT > 0, and DS > 0 for both control and intervention groups at six-month follow-up but this increase was significantly higher for the control than intervention group (*P* < .001). Among all variables included in the multiple logistic regression model, just being in the intervention group showed a significant effect in which children in the control group had a 4.2-fold (95% CI: 2.36-7.54) greater chance for developing new caries than children in the intervention group. There was a statistically significant increase in the percentage of children with favourable levels of behaviors between baseline and 3-month follow-up (*P* < .05, *P* < .001).

**Conclusion:**

Providing access to oral health services such as oral examination, fluoride varnish application, and oral health education to reduce dental caries and improve oral health practices seems to be effective among primary schoolchildren.

## Introduction

Oral diseases, which are widely prevalent non-communicable diseases, represent significant public health concerns on a global scale.^1^ Studies have shown that dental caries affect a substantial proportion of schoolchildren and adults in low- and middle-income countries, with estimates ranging from 60% to 90%.^2^ Lack of knowledge pertaining to preventive measures, restricted access to oral health services, high consumption of sugars, and insufficient exposure to fluoride are correlated with an escalated likelihood of dental caries.^3^^,^^4^ The majority of these risk factors are behavior and lifestyle-related and can be avoided through the promotion of oral hygiene and oral health education (OHE).^4^ Schools present an opportunity for the promotion of oral health as children spend the majority of their time there. Moreover, schools establish connections to the community, families, and healthcare providers which facilitate conducting oral health promotion programs. These programs can enhance the oral health of children by addressing risk factors for oral diseases at an early stage of life.^5^, ^6^, ^7^

School-based programs for promoting oral health offer numerous benefits including the ability to reach a diverse population of children, establish connections with their families and communities, enhance the accessibility of dental care (particularly for disadvantaged children), and engage with an age group that is receptive to adopting positive health-related beliefs, attitudes, and behaviors.^8^ The World Health Organization (WHO) recommended in 2002 that schools be utilized as a backdrop for interventions aiming at oral health promotion.^9^ Within school programs, several activities can be implemented, including oral health education, supervised tooth brushing, and the application of fluoride and fissure sealants.^10^ Various examples from the literature demonstrate the positive impact of school-based oral health programs in reducing the burden of oral disease among primary school children in countries with low and middle incomes,^11^ the caries level of primary school children,^12^, ^13^, ^14^ as well as the improvement in children's oral health behavior.^15^^,^^16^

Despite the high prevalence of dental caries among schoolchildren in Iraq, with rates of 61% and 84% for primary and permanent dentitions, respectively,^17^ there is a lack of information regarding the effectiveness of school oral health programs on dental caries. The purpose of this research was to assess the impact of a school-based oral health promotion program on the occurrence of dental caries in permanent teeth among 8-10-year-old children in Iraq. Our hypothesis was that the combined use of fluoride varnish and oral health education would be more effective in reducing caries compared to oral health education alone and that oral health education would lead to improved oral health behaviors in children.

## Materials and methods

### Study design and sampling issues

**Fig. 1:**
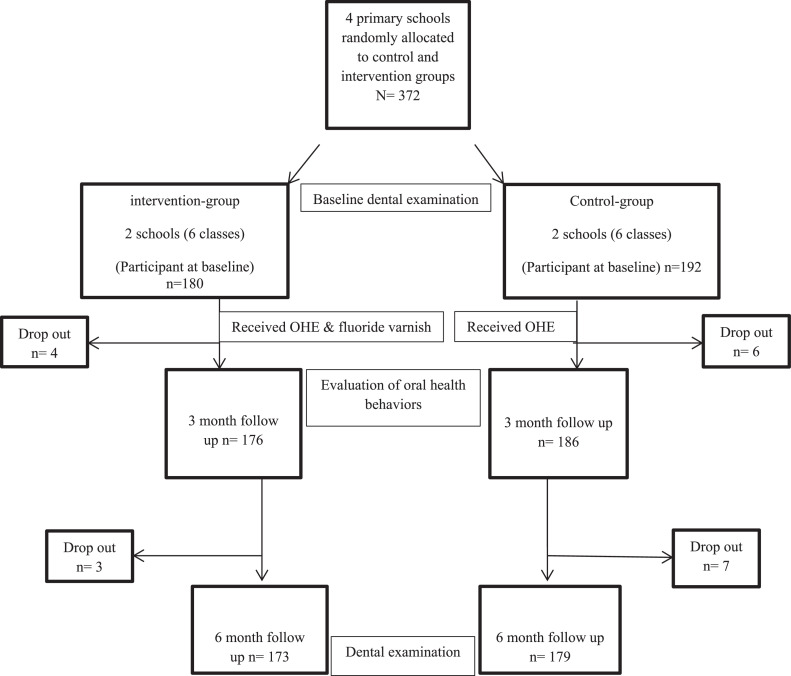
Flowchart of the study.

This study employed a cluster randomised controlled parallel trial design, which took place between October and May 2023. The target population consisted of children between the ages of 8 and 10 who attended elementary schools in Kut City, Iraq. To determine the appropriate sample size, the researchers referred to a recent study that reported a mean DMFS difference of 2.8 units between two groups.^12^ Based on this information, a minimum of 128 children per group was deemed necessary. To account for potential loss-to-follow-up, a 40% increase was applied, resulting in at least 180 children per group. Given that the sampling method in this study was based on schools (as clusters) and the uneven distribution of students in each class within the chosen schools, we ultimately obtained a sample size of 192 students in the control group and 180 students in the intervention group. The inclusion criteria encompassed individuals of Iraqi nationality who were between the ages of 8 and 10. The exclusion criteria consisted of individuals with any systemic illness, disabilities, or those who failed to provide consent for participation in the study. During the subject recruitment process, two girls' and two boys' schools (as clusters) were selected randomly from a comprehensive list of 40 elementary schools within the city. This selection was carried out utilising a lottery method by an assistant who did not take part in the main study. Subsequently, these four schools were randomly assigned (one girls' school and one boys' school) to either the intervention or control group based on the results of a coin flip. In order to identify the study subjects, three classes (one from the 3rd grade, one from the 4th grade and one from the 5th grade) were randomly chosen for each of the control and intervention schools. The selection of these classes was carried out by school administrators using a simple randomisation technique, whereby the classes related to each grade were assigned random numbers and then allocated to either the intervention or control group. The total number of students who underwent examination in each school is presented in Table 1. Furthermore, Figure 1 depicts the recruitment process.

**Table 1:** Baseline characteristics of the children in control (*n* = 192) and intervention (*n* = 180) groups.

		Control	Intervention	*P* value*
		*n* (%)	*n* (%)	
Age (years)	8	60 (31%)	62 (34%)	.276
	9	57 (29%)	62 (34%)	
	10	75 (40%)	56 (32%)	
Gender	Boys	101 (53%)	90 (50%)	.616
	Girls	91 (47%)	90 (50%)	
Father education level	Primary school	30 (15%)	19 (11%)	.131
	Secondary or high school	68 (35%)	55 (30%)	
	Graduate	94 (50%)	106 (59%)	
Mother education level	Primary school	30 (15%)	28 (15%)	.189
	Secondary or high school	74 (38%)	54 (30%)	
	Graduate	88 (47%)	98 (55%)	
Mother age (years)	< 40 years	118 (61%)	124 (68%)	.133
	≥ 40 years	74 (39%)	65 (32%)	
Brushing frequency	Irregular	28 (15%)	32 (17%)	.652
	Once a day	105 (55%)	87 (48%)	
	≥ Twice per day	59 (30%)	61 (35%)	
Sweet snacks between meals	< Once a day	14 (7%)	12 (6%)	.162
	Once a day	49 (25%)	63 (35%)	
	≥ Twice per day	129 (68%)	105(59%)	
Past dental visit	Never	90 (47%)	91 (50%)	.634
	Last year and before	88 (46%)	73 (40%)	
	During last six month	14 (7%)	18 (10%)	
Caries experience	Mean DMFS (SD)	2.5 (3.43)	2.2 (3.42)	.283⁎⁎

⁎ Statistical evaluation by the Chi-square between control and intervention groups.

⁎⁎ Statistical evaluation by the Independent sample t-test between control and intervention groups.

### Ethical issues and calibration process

The Ethics Committee of Shahid Beheshti School of Dentistry approved this study (IR.SBMU.DRC.REC.1401.030), and the trial was registered in Iranian Registry of clinical Trial (IRCT2022062705529N1). Permission was acquired from the General Directorate of Education of Kut City as well as the chosen school principals. In each classroom, one of the authors (HA) elucidated the purpose of the study to the students and gave them both the questionnaire and the written consent form to be taken to their home. They were instructed to deliver the questionnaire and consent form to their parents to be completed, signed, and returned to their schools in the subsequent days. In the event that inquiries arose regarding the content of the questionnaire, HA provided additional clarification to both the children and their parents. The clinical oral examination in this study was performed by a single examiner (HA) who had been calibrated for recording ICDAS. Furthermore, in order to be sure about the reproducibility of the process, the examiner (HA) conducted oral examination on a group of 20 children twice with a two-week interval resulting in a weighted kappa coefficient of >0.8 for intra-examiner agreement.

### Data collection process

On each working day, a group of five children was brought to the examination room provided by the school authority. Following the collection and verification of signed consent and completed questionnaires, an oral examination was administered using 0.5 mm ball-end WHO probes (Dentirak®) and disposable mouth mirrors while the child was seated on a mobile dental chair under the artificial light of dental unit.

### Data collection tools and study variables

The questionnaire employed in this study included demographic characteristics of the students (age, gender, parents' age and education) and, questions related to the students' oral health-related behaviors (frequency of brushing, using fluoridated toothpaste, frequency of flossing, eating sugary snacks, time and reason for the last dental visit). Later in the analysis, favourable level of behaviours was defined as tooth brushing ≥ twice per day, using fluoridated toothpaste, floss at least once a day, eating sweet snacks less than once a day, dental visit within last six months, and dental visit for checkup. The Clinical assessment involved documenting the caries condition based on the International Caries Detection and Assessment System (ICDAS).^18^ This system utilizes a two-digit code to identify primary caries. The first digit indicates tooth restoration, ranging from 0 to 8, while the second digit represents caries, ranging from 0 to 6. Additional codes from 96 to 99 also indicate missing conditions. Before the oral examination, participants were advised to brush their teeth. Subsequently, tooth surfaces were cleaned and dried using water and an air syringe, following which the caries status was documented utilising the ICDAS coding system. In order to facilitate meaningful comparisons with previous studies of a similar nature, the ICDAS codes were employed in this study to calculate DMFS and DMFT.^19^, ^20^, ^21^ Specifically, codes 3-6 from the second digit were designated as decayed (D), codes 97 as missed (M), and codes 3-5 from the first digit as filled (F). It is important to mention that the detection of dental caries within a six-month period can be achieved considering the sensitivity of ICDAS in identifying early signs of tooth decay.^22^^,^^23^

### Intervention

At the beginning of the study, subjects in the intervention group received oral health education and a single dose of 5% sodium fluoride varnish (Clipro™, white varnish, 3M ESPE, Saint Paul, MN, USA) for all teeth surfaces while the control group only received oral health education. Topical application of fluoride varnish was performed by HA. According to a guideline for fluoride varnish application in school settings,^24^ after brushing and confirmation of the cleanliness of the teeth, they were subsequently isolated using cotton rolls and dried with gauze pads prior to the application of 0.5 ml of fluoride varnish on all tooth surfaces using a single-use brush. In order to achieve the optimal effect of the varnish, the children were instructed to avoid drinking or eating for half an hour, chewing hard foods for 4 hours and not to brush their teeth until the next day.^25^ HA provided oral health education for the students in both groups while their teacher was present in each class. These education sessions included the proper tooth brushing technique (modified bass technique) that was demonstrated on a plastic model, the importance of brushing at least twice a day (in the morning and before bed time at night) emphasis on at least two minutes brushing time, proper use of dental floss at least once a day, having appropriate diet (drinking water instead of sugary beverage, avoiding sugary foods as much as possible or limiting to main mealtimes, eating fruits and vegetables), following regular dental visit, and emphasis on washing mouth after each eating. Besides the face-to-face education, the students were provided with an oral health educational pamphlet including the above-mentioned contents and a free goody bag containing a toothbrush, a fluoride containing dentifrice with 1450 ppm fluoride, and interdental floss. After three months from the baseline evaluation, HA visited the schools to collect data regarding students’ oral health-related behaviours by means of the same questionnaire. The second follow-up evaluation took place after 6 months from the baseline evaluation which included the clinical examination by HA for all students in both groups.

### Outcome data

The primary outcome data in this study were caries increment in the permanent teeth (changes in DMFS and DMFT scores after 6 months), caries prevalence (children with caries/all children × 100), and caries incidence (children with new caries/all children × 100) after six months. The development of a newly decayed surface was assessed by calculating the difference between the average score of decayed surfaces at the end of a 6-month period (final DS) and the average score of decayed surfaces at the start of the study (initial DS). A child was deemed an incident case of decayed surface if the difference between the final DS and the initial DS was greater than zero. The secondary outcome data was any change in oral health behaviors in the students of both groups after 3 months.

### Statistical analysis

Statistical analysis included the chi-square test (to compare the sociodemographic and oral health behavior variables, and new decayed inside and between the two groups), the independent t-test (to compare the mean scores of caries experience and caries increment inside and between the two groups), the McNemar test (to compare the frequency of oral health behaviors before and after intervention in both groups), and a multiple logistic regression model (to identify the variables associated with developing new caries as a binary variable with 0 denoting no newly caries developed and 1 having newly developed caries). For all statistical analyses, significance was considered at *P* < .05. The data were analysed using the IBM SPSS program version 25 (Armonk®, NY).

## Results

The study population of this research comprised 372 children. The baseline characteristics of these children are presented in Table 1. As indicated in the table, the participants were evenly distributed across the various subgroups, with comparable proportions and no statistically significant difference between the intervention and control groups.

Table 2 shows percentage of children's oral health behaviors at baseline and 3-month follow-up. In both groups, there was a statistically significant increase in the percentage of children with favourable level of behaviors between baseline and 3-month follow-up. There were no significant differences in favourable oral health behaviors between both groups at baseline, and 3 months.

**Table 2:** Percentage of children with favourable oral health behaviors at baseline and 3-month follow-up.

	Control (*n* = 186)			Intervention (*n* = 176)			Compare 2 groups at baseline	Compare 2 groups at follow up
	Baseline	Follow up	*P* value*	Baseline	Follow up	*P* value*	*P* value$	*P* value$
Tooth brushing ≥ twice per day	30.6	37.0	< .001	30.1	35.2	.004	.912	.711
Using fluoridated toothpaste	30.1	43.0	< .001	28.9	43.1	< .001	.814	.787
Floss at least once a day	8.0	12.9	.002	7.3	11.9	.008	.809	.667
Eating sweet snacks less than once a day	7.5	11.2	.016	6.2	10.7	.008	.632	.881
Dental visit within last six months	7.5	27.9	< .001	10..2	23.8	< .001	.336	.963
Dental visit for check up	8.6	14.5	.019	6.8	11.9	.022	.525	.386

⁎ Statistical evaluation by the McNemar test between baseline and follow up in both groups.

$ Statistical evaluation by the Chi-square test between control and intervention groups.

Caries experience, prevalence, and percentages of children with new decayed tooth surfaces are demonstrated in Table 3. As it appears in the table, an increase is evident in the mean scores of DMFS, DMFT, number of children with DMFT > 0 and DS > 0 for both control and intervention groups at six-month follow-up but this increase was significantly higher for the control than intervention group.

**Table 3:** Mean DMFS, mean DMFT, percentages of children with DMFT > 0 and DS > 0 at baseline and 6-month follow-up.

Mean DMFS (±SD)	Control	Intervention	*P* value*
	*n* = 179	*n* = 173	
Baseline	2.4 (3.26)	2.2 (3.41)	.513
Follow up	3.2 (3.67)	2.4 (3.47)	.039
Difference (increment)	0.8 (1.59)	0.2 (0.77)	< .001
Mean DMFT (±SD)			
Baseline	1.4 (1.66)	1.3 (1.52)	.456
Follow up	1.9 (1.86)	1.4 (1.56)	.008
Difference (increment)	0.5 (0.74)	0.1(0.93)	< .001
Number of children with DMFT > 0 (%)			
Baseline	107 (59.7)	105 (60.7)	.860⁎⁎
Follow up	130 (72.5)	109 (63.0)	.041⁎⁎
Difference (incidence)	23 (12.8)	4 (2.3)	< .001⁎⁎
Number of children with DS > 0 (%)			
Baseline	105 (58.6)	104 (60.1)	.781⁎⁎
Follow up	129 (72.0)	108 (62.4)	.048⁎⁎
New decayed surface (incident case)	63 (35.1)	24 (12.1)	< .001⁎⁎

⁎ Statistical evaluation by the Independent sample t-test between control and intervention groups.

⁎⁎ Statistical evaluation by the Chi-square test between control and intervention groups. DS = decayed surfaces.

**Fig. 2:**
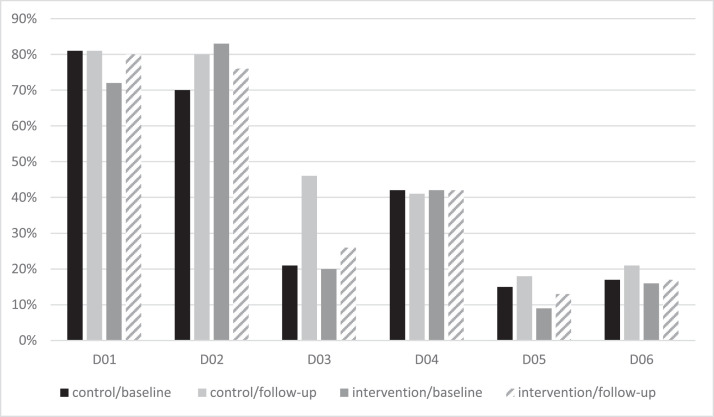
Percentages of children with ICDAS codes at baseline and 6 months follow-up separately for the intervention and control groups.

Figure 2 illustrates the distribution of children according to ICDAS scores. As it appears in the figure, a statistically significant difference was found between intervention and control groups at baseline (*P* = .004) in code D02 and also at 6 months follow-up (*P* < .001) in code D03.

Table 4 presents the result of two logistic regression models for the evaluation of factors impacting the development of new caries in children's teeth at 6-month follow-up. Among all variables included in the models, just being in the intervention group showed a significant effect in which children in the control group had a 4.2-fold (95% CI: 2.36-7.54) greater chance for developing new caries than children in the intervention group. No side effects or adverse events were reported by children during the six months of the study.

**Table 4:** Determinants for the development of new caries at 6-month follow-up as assessed by simple and multiple binary logistic regression models with simultaneous control of confounders.

Variables	Crude OR	95% CI	*P* value*	Adj. OR*	95% CI	*P* value*
Demographic factors						
Age (reference: 8 years)						
9 years	1.55	(0.83, 1.94)	.164	1.75	(0.87, 3.52)	.115
10 years	1.59	(0.85, 1.99)	.145	1.76	(0.89, 3.46)	.102
Gender (reference: male)	1.41	(0.86, 2.32)	.168	1.25	(0.72, 2.18)	.418
Mother's age (reference: ≥ 40 year)	1.06	(0.63,1.78)	.818	1.25	(0.70, 2.22)	.448
Father's education (reference: graduate)						
Primary school	2.00	(0.98, 4.11)	.057	2.29	(0.94, 5.57)	.066
Secondary/high school	1.20	(0.70, 2.08)	.496	1.31	(0,67, 2.56)	.417
Mother's education (reference: graduate)						
Primary school	1.07	(0.51, 2.24)	.844	0.91	(0.37, 2.27)	.853
Secondary/high school	1.25	(0.73, 2.15)	.400	0.92	(0.47. 1.80)	.825
Oral health behaviors at 3 months						
Tooth brushing ≥ twice per day (reference: yes)	1.07	(0.64, 1.80)	.783	1.18	(0.67, 2.08)	.550
Eating sweet snacks less than once a day (reference: yes)	1.20	(0.52, 2.72)	.663	1.44	(0.85, 3.55)	.422
Dental visit within last six months (reference: yes)	0.61	(0.35, 1.06)	.082	0.60	(0.33, 1.12)	.111
Baseline DMFS score	1.03	(0.96, 1.10)	.382	1.03	(0.95, 1.12)	.407
Intervention group (reference: yes)	3.93	(2.26, 6.81)	< .001*	4.20	(2.36, 7.54)	< .001*

⁎ Adjusted ORs are given by adjusting the effect of rest of variables in multiple logistic regression

## Discussion

Findings of the present study showed a positive impact of the intervention in which the percentages of the children with favourable oral health behavior due to OHE were increased in both groups. Moreover, mean of dental caries indicators showed an increase in both groups in the follow up examination but this increase was more evident in the control group.^9^ Health education is a prerequisite for behavior change, favourable health status, and health promotion. The effectiveness of OHE in the promotion of individuals’ oral health status, knowledge, and attitude has been documented.^26^

Positive impact of OHE on the oral health behaviors of schoolchildren in the present study is in line with the findings of similar studies^16^^,^^27^^,^^28^ that provide further clues regarding the effectiveness of OHE in the promotion of oral health. Furthermore, this simple school-based intervention may be adopted by regional authorities as it has been emphasised by WHO as a key strategy for oral health promotion of schoolchildren.^29^

Adherence to optimal oral self-care among these schoolchildren, however, was non-prevalent since around one-third of them reported more than once a day toothbrushing and dental visit within last year. In contrast, more than fifty percent of adolescents in some European countries reported toothbrushing on at least twice per day basis^30^ and having a dental visit during last year.^31^

The prevalence dental caries in this study (61%) is in accordance with previous investigations carried out in Iraq^32^ and the Eastern Mediterranean Region^33^ but higher than what reported form similar studies in Iran (41%),^34^ China (21%),^35^ and Italy (45%).^36^ On the other hand, results from implementation of the intervention in this study indicated a positive impact on the occurrence and prevalence of dental caries among school children. These results align with the outcomes of a study conducted in China,^13^ which employed a similar methodology on primary school children. The effectiveness of fluoride varnish in reduction of caries prevalence is well established as a Cochrane systematic review revealed that the use of fluoride varnish was associated on average with a 43% reduction in DMFS.^37^ Moreover, the efficacy of fluoride varnish application for caries control among schoolchildren has been acknowledged in several studies.^12^, ^13^, ^14^^,^^38^ Our results revealed that fluoride varnish was more effective in caries reduction than basic oral hygiene practice, consistent with previous report in Australia^39^ using the same health indicator (DMFT).

In line with these studies, findings of the present investigation, showed a beneficial impact of fluoride varnish application on reducing the mean scores of caries increment in the permanent dentition. The results of this study are expected to provide an overview for designing oral health intervention programs among primary schoolchildren that can be implemented in middle and low-income countries, particularly, in Iraq where dental caries is still highly prevalent in primary (84%) and permanent (61%) dentitions.^17^

In this specific study, it is important to acknowledge the possibility of an observer bias due to the inability to blind the examiner with regards to the intervention group and the control group. Since the examination of the students took place within the school premises, the examiner was aware of which schools were assigned to the intervention group. This particular circumstance poses a limitation in the implementation of the study. However, intra-examiner reliability (Kappa: > 0.8) suggests a good consistency for the examination procedure. On the other hand, there were several strengths to this study. To the best of our knowledge, this trial stands as the first attempt to evaluate the effectiveness of a school-based oral health preventive/educational program among children aged 8-10 years in Iraq. The use of random sampling techniques ensured that the selected children were representative of the population, enhancing the generalizability of the findings to school children in Kut City, Iraq. The inclusion of teachers in oral health education sessions was beneficial as they have regular contact with students. Furthermore, the rate of loss to follow-up in the study was relatively low. Lastly, the study employed a multiple logistic regression model that assessed the effect of the intervention while controlling of accounted for sociodemographic factors, oral health behaviours, and baseline caries experience on caries development.

## Conclusions

This study indicated the effectiveness of fluoride varnish application on reducing the incidence of dental caries in permanent dentition and OHE in increasing the level of favourable oral health practices among the studied children. This evidence will help policy makers for allocating the scarce resources for oral health promotion of primary schoolchildren.

## Conflict of interest

The authors have no conflicts of interest.

## Appendix Supplementary materials

Image, application 1

Image, application 2
