# Supplementary material for: Effectiveness of a School-Based Oral Health Promotion Program on Dental Caries Among Iraqi School Children: A Cluster Randomised Controlled Trial
Source: Int Dent J. 2024 Sep 24;75(2):744–51. doi: 10.1016/j.identj.2024.07.1214 (PMC11976541; doi:10.1016/j.identj.2024.07.1214)
Supplement: Supplementary file 2 [file mmc2.pdf]

Shahid Beheshti University of Medical Sciences

Research Institute of Dental Sciences-Shahid Beheshti University of Medical Sciences

Research Ethics Committees Certificate

Approval ID:	IR.SBMU.DRC.REC.1401.030	Approval Date:	2022-05-24
Evaluated by:	Research Ethics Committees of Research Institute of Dental Sciences-Shahid Beheshti University of Medical Sciences		
Status:	Approved		
Approval Statement:	The project was found to be in accordance to the ethical principles and the national norms and standards for conducting Medical Research in Iran.<br>Notice:<br>1. Although the proposal has been approved by the Biomedical Research Ethics Committee, meeting the professional and legal requirements is the sole responsibility of the PI and other project collaborators.<br>2. This certificate is reliant on the proposal/documents received by this committee on 2022-05-24. The committee must be notified by the PI as soon as the proposal/documents are modified.		
Thesis Title:	Oral health related quality of life and comparison between two preventive regimens among Iraqi preadolescents		
Supervisor:	Name: Hadi Ghasemi<br>Email: ha.ghasemi@sbmu.ac.ir		
Student:	Name: hanan Al autry<br>Email: hananfadhil@uowasit.edu.iq		

Dr. Seyed Mohammadreza Safavi
Committee Director
Research Institute of Dental Sciences-Shahid Beheshti University of Medical Sciences

Dr. Mohammad Jafar Eghbal
Committee Secretary
Research Institute of Dental Sciences-Shahid Beheshti University of Medical Sciences
